# Effectiveness of cold vibratory stimuli on pain perception governing infiltration anesthesia in the maxillary arch in children: a randomized controlled clinical trial

**DOI:** 10.1186/s12903-025-06170-4

**Published:** 2025-06-03

**Authors:** Hoda Mohamed Hambouta, Dina Aly Sharaf, Nadia Aziz Wahba

**Affiliations:** Pediatric Dentistry and Dental Public Health Department, Faculty of Dentistry, Alexandria University, Alexandria, Egypt

**Keywords:** Buzzy^®^, Palatal injection, Intrapapillary injection, Buccal injection, Maxillary infiltration anaesthesia, Pain management

## Abstract

**Background:**

Buzzy^®^ - a vibratory unit with ice wings -was introduced to reduce pain induced by needle prick. Objective of the study was to evaluate the effectiveness of “Buzzy^®^” in reducing pain perception during administration of maxillary infiltration local anaesthesia compared to the traditional topical anaesthetic gel in pediatric patients. In addition, it aimed to evaluate its effectiveness in intrapapillary anaesthesia in comparison to the conventional palatal injection following buccal infiltration anaesthesia.

**Methods:**

Randomized controlled clinical trial with parallel arms. Forty-eight cooperative children aged 6–8 years indicated for maxillary first or second primary molar extractions were selected. They were randomly allocated into 2 main groups (I and II) then into 2 subgroups each (A and B). All groups received buccal infiltration anaesthesia. Group I received local anaesthesia following the use of Buzzy^®^ extra orally for 2 min. Group II received local anaesthesia following topical anaesthetic gel (benzocaine 20%) intraorally for 1 min. Subgroups A received routine palatal injection whereas subgroups B received intrapapillary anaesthesia. Each patient was videotaped during local anaesthesia injection to objectively assess the reaction using sound, eye, motor scale (SEM). After local anaesthesia administration, pain was subjectively assessed using the visual analogue scale (VAS). Normality was checked in age and VAS using Shaprio Wilk test. The Independent T test was used to compare age between groups. Differences in SEM and VAS between buzzy and infiltration groups were analyzed using the Mann Whitney U test while Wilcoxon sign Rank test was used to analyze the difference in SEM and VAS between intrapapillary and palatal injection within each group. All tests were two tailed and the significance level was set at *p* value ≤ 0.05.

**Results:**

No statistical significance was observed in pain perception between Buzzy and topical gel regarding buccal local anesthesia. Conversely, objective and subjective statistical significance in pain perception between (Group I) and (Group II) regarding both palatal and intrapapillary anesthesia was recorded.

**Conclusion:**

Applying external cold and vibratory stimulant can reduce pain perception during maxillary buccal, intrapapillary and palatal local infiltration anesthesia.

**Clinical trial registration:**

The clinical registration number in ClinicalTrials.gov holds the identifier: NCT05857033 retrospectively registered on 12/5/2023.

**Supplementary Information:**

The online version contains supplementary material available at 10.1186/s12903-025-06170-4.

## Background

Absence of pain in dental procedures is the ultimate goal that dentists seek. Perception of pain in dental practice is the outcome of a combination of objective and subjective factors. Both vary and contribute to the individual nature of pain [1]. Pain management and prevention rely heavily on local anaesthetics. Although its purpose is to make dental procedures painless, fear of the needle puncture prevents children to seek dental treatment [2]. 

In this context, dentists applied a variety of techniques to minimize discomfort when delivering local anaesthesia (LA), such as applying topical anaesthetics, pointing the needle bevel in the right direction, using a small-gauge needle, injecting the anaesthetic agent slowly and warming or buffering the anaesthetic agent [3]. Other factors are audio visual distraction, hypnosis, computer assisted anaesthesia system and counter-stimulation/ distraction technique [4]. 

The gate control theory of pain- first presented in 1965 by Melzack and Wall [5]- proposes that a mechanism in the dorsal horns of the spinal cord functions as a gate. It either inhibits or facilitates transmission of pain from the body to the brain. Accordingly, pain can be alleviated by stimulating nerve fibers that convey non-noxious inputs. The idea holds that stimulation of the larger diameter fibers can seal a brain gate to nociceptive inputs, hence reducing the feeling of pain with the proper amounts of coolness, warmth, rubbing, pressure, or vibration [6]. 

Many studies with different devices or techniques were conducted to check the validity of the gate control theory. DiFelice et al. [7] concluded that a vibratory device and topical anaesthetic in combination showed less pain perception than topical anesthetic alone. Topical gels are useful, although the patient’s taste and smell reactions of the topical gel could have unfavorable effects [8, 9]. Another method for diverting the patient’s attention is through touch, visual, and auditory stimuli. A simple distraction technique is to manually shake the patient’s cheek. It can be successful, but results may vary [8]. 

Shilpapriya et al. [10] revealed that Dental vibe – an intraoral device that provides vibrations at the dental injection site- can be a useful technique for reducing discomfort while administering local anaesthesia. Additional distraction is produced by the vibration’s sound. Moreover, the vibration speeds up the effect of the anaesthetic by dispersing it after dental injection [11]. Alanzi et al. [12] and Bilsin et al. [13]revealed favorable outcomes from Buzzy that were further demonstrated in a systematic review and meta-analysis by Faghihian et al. [14]. The author concluded promising outcomes in reducing pain perception in dental injections using Buzzy, unlike the Dental Vibe.

Buzzy^®^ is a device that was developed to primarily enable pain- free vaccinations for children [15]. The device was then used in a variety of medical research [16–18]. The theory that pain is psychological in nature and depends on the patient’s attention and perception underlies the use of cold (temperature) in combination with vibration (stimulation). It has been suggested that devices with vibration produce a distracting setting, leading the brain to predominantly transmit the vibrations, thus permitting LA to flow without pain. The inclusion of the cold factor further distorts how the pain pathway interprets signals [12]. 

The aim of this research was to evaluate the effectiveness of a vibratory unit with ice wings “Buzzy^®^” in terms of pain perception during maxillary LA administration, i.e., buccal, intrapapillary and palatal compared to the traditional topical anaesthetic gel in pediatric patients. Additionally, it aimed to evaluate its effectiveness in intrapapillary anaesthesia in comparison to the conventional palatal injection following buccal infiltration anaesthesia. The null hypothesis adopted was that there would be no significant difference in pain perception between Buzzy and conventional topical anesthesia during maxillary buccal, and either intrapapillary, or palatal infiltration anesthesia in children.

The rationale for this study lies in the need to optimize pain management during local anesthesia. There is a lack of comprehensive, direct comparison between palatal and intrapapillary injections. Existing studies have often focused only on one injection site (either buccal, palatal or nerve block), without examining the impact when using more than one type within the maxillary arch. The lack of comprehensive evidence leaves a gap of knowledge, as to which method is most effective among different injection types (buccal, palatal, and intrapapillary). Improving pain management strategies during injections is vital for ensuring positive dental experiences and encouraging continued care. In this context, understanding which technique—Buzzy or topical anesthetic gel—more effectively reduces pain perception and is more beneficial for specific injections is crucial.

## Methods

This study was a randomized controlled clinical trial with parallel arms. Forty-eight patients were selected for maxillary primary molar extractions from the outpatient clinic of Pediatric Dentistry and Public Health Department, Faculty of Dentistry, Alexandria University, Egypt after securing necessary consents.

Sample size was estimated assuming 5% alpha error and 80% study power. According to Bilsin et al. [13], the mean (SD) pain score was 3.333 (1.917) and 0.867 (1.136) for children anesthetized without cold vibration stimuli and children anesthetized with cold vibration stimuli. Based on difference between two independent means using the highest SD = 1.917 to ensure enough study power, the sample size was to be 12 patients per group. Total sample size = number per group x number of groups x number of subgroups = 12 × 2 × 2 = 48 patients. The sample size was based on Rosner’s method [19] calculated by G*Power 3.1.9.7 [20]. The effect size was 1.286.

## Inclusion and exclusion criteria

Children aged 6–8 years old, physical status American Standards Association (ASA) I or II [21] (Additional File 1), with one maxillary primary molar indicated for extraction, were chosen following a full clinical evaluation. Participants scored 3 or 4 on Frankl behavioural rating scale indicating cooperation were selected [22]. (Additional File 2) Teeth with signs of pathosis, or acute oral orofacial infection that could affect anesthetic assessment were not included in the study. No participants with known allergy to local anesthesia or any of its components were selected.

Participants were randomly allocated into two main groups (I & II). Each group was categorized into two subgroups (A and B) according to the pain reduction technique used before LA administration. Test group (I) used Buzzy, whereas control group (II) used topical anesthetic gel. Subgroups A received buccal and routine palatal injection whereas subgroups B received buccal and intrapapillary anaesthesia.

Parents who consented were fully informed about the purpose and procedures of the study, as well as its benefits and risks. Informed consent was obtained both orally and in writing before participation and publication. Participants were randomly assigned using a computer-generated list of numbers to one of the four subgroups [23]. To ensure allocation concealment, an independent personnel was delegated to give each child a serial number written in identical sheets of paper with the group to which each child is allocated, then place the sheets inside opaque envelopes carrying their respective names. The personnel collected the envelopes and only opened them at the time of the LA injection. Due to the difference between the used techniques the operator was not blinded. However, the statistician and the participants were blinded to the treatment groups. Thus, the study was single-blinded. Each clinical procedure was carried out by a single operator for the purpose of standardization. The operator was trained and calibrated to assess pain using Sound Eye Motor scale (SEM). Buzzy^®^ was used on ten patients prior to LA administration and extraction then classified the child’s behavior on videotapes. The exercise was repeated after a 7-day gap in order to establish an acceptable degree of examiner reliability. Kappa test yielded a score of 0.871, *p* value < 0.0001*, which ensured excellent agreement.

This research was conducted following the Declaration of Helsinki and approved by the Research Ethics Committee of Alexandria University, the Faculty of Dentistry. (#0568 − 12/2022) (IRB No. 00010556 - IORG 0008839).

The clinical work was conducted from January 2023 to April 2023. Preliminary screening and full medical and dental history were carried out to select patients who fulfilled the inclusion criteria. All participants had previous dental history with local anaesthesia experience. Parents and children were given age-appropriate oral hygiene instructions. Patient’s body weight was recorded to calculate the recommended dose of anaesthesia. For the test group, with a mean weight of 23 kg, the maximum recommended dose of articaine is 2 cartridges (or 3.6 ml). In the control group, for a weight of 26 kg, the maximum recommended dose is 2.5 cartridges (or 4.5 ml). However, it is important to note that in the present study, only one cartridge (1.8 ml) was needed to achieve profound anesthesia for all participants. A signed informed consent was obtained from the guardian before the treatment, and the child granted verbal consent prior to the intervention.

For participants in subgroups IA and IB (experimental group), a demonstration of Buzzy^®^ (Pain Care Labs, a dba of MMJ Labs, 195 Arizona Ave NE LW08, Atlanta, GA 30307) was performed by explaining that their teeth will fall asleep after placing the honeybee-like vibration unit and the ice wings to remove all the offending germs. The ice wings were frozen at -18 °C. The wings were then attached to the vibrating unit and inserted in a nylon bag to ensure infection control measures. Tell show do was adopted, where participants were asked to try it on the hands before applying it to the face. The device was placed against the zygomatic arch and switched on for two minutes after which LA was administered. (Additional File 3) A standardized 1.8 ml LA cartridge of Articaine 40 mg/0.01 ml with epinephrine 1:100.000 (ARTINIBSA, Inibsa Dental S.L.U, 08185 Lliçà de Vall, Barcelona, Spain) was injected using a 30-gauge dental needle. Both subgroups received buccal infiltration followed immediately by palatal anesthesia. As for the palatal tissues, subgroup IA received palatal infiltration, whereas subgroup IB received intrapapillary infiltration during which the palatal mucosa blanched.

The patient was informed that the procedure would be videotaped to document good behavior. It was later used for SEM evaluation.

For participants in subgroups IIA and IIB (control group), anesthetic topical gel − 20% benzocaine (Dharma Iolite Research Inc, Miami, Florida)- was introduced by explaining that their teeth will fall asleep after placing the strawberry jelly to remove all the germs. A gauze (2 inch x2 inch) was applied to dry the buccal tissues to improve gel absorption that was placed for one minute. In subgroup IIA topical gel was applied at the buccal and palatal infiltration site, whereas in subgroup IIB topical gel was applied at the buccal infiltration site only. Saliva ejector was used during the administration of the topical anaesthesia. The procedure was videotaped for further SEM evaluation.

Extraction was performed in both groups, following the guidelines of the American Academy of Pediatric Dentistry. Participants received post-extraction instructions. Planning for space maintenance was considered where necessary.

Pain was measured objectively using the sound-eye-motor (SEM) scale [24]. (Additional File 4) Subjective assessment following LA administration was rated by the patient using a visual analog scale (VAS). (Additional File 5) The 10 cm VAS scale ranged from “no pain” (smiley face = 0) to “unbearable pain” (frowning face = 10) [25] was categorized into three levels: no pain (VAS = 0), mild pain (VAS = 1–3), moderate pain (VAS = 4–6), and severe pain (VAS = 7–10) [26]. 

Twenty-four hours post extraction, parents were reached through a telephone call to determine if there was any lip/cheek biting or any negative events during the recovery process.

Data was analyzed using IBM SPSS version 23, Armonk, NY, USA. Normality was checked in age and VAS using ***Shaprio Wilk test***. Age was normally distributed, and it was presented using mean and standard deviation. SEM and VAS were not normally distributed thus both were presented mainly using median, 95% confidence interval, minimum and maximum values. The ***Independent T test*** was used to compare age between groups. Differences in SEM and VAS between buzzy and infiltration groups were analyzed using the ***Mann Whitney U test*** while ***Wilcoxon sign Rank test*** was used to analyze the difference in SEM and VAS between intrapapillary and palatal injection within each group. All tests were two tailed and the significance level was set at *p* value ≤ 0.05.

All procedures were performed in compliance with the Helsinki Declaration and its later amendments. Reporting of the study followed (CONSORT) checklist [27]. 

The clinical registration number in ClinicalTrials.gov holds the identifier: NCT05857033 posted on the 12th of May 2023.

## Results

Fifty-five children were screened for participation and assessed for eligibility, among which forty- eight were enrolled in the study. None of them discontinued participation or was lost to follow up.

The mean and standard deviation of the weight (kg) in the test group was 26.00 ± 4.16 and in the control group 23.58 ± 3.651 with no statistical difference. As for the height (cm), the mean and standard deviation in the test group was 126.67 ± 7.46 and in the control group 122.00 ± 7.93 with no statistical difference.

The study group I (Buzzy) included 13 boys and 11 girls, with a mean age 7.60 ± 1.43 years, whereas the control group II (topical anaesthetic gel) consisted of 14 boys and 10 girls, with a mean age 7.10 ± 1.15. There was no statistical significance neither in sex nor age in both study groups. The consort flow diagram is shown in conjunction with the consort checklist.

**Fig. 1:**
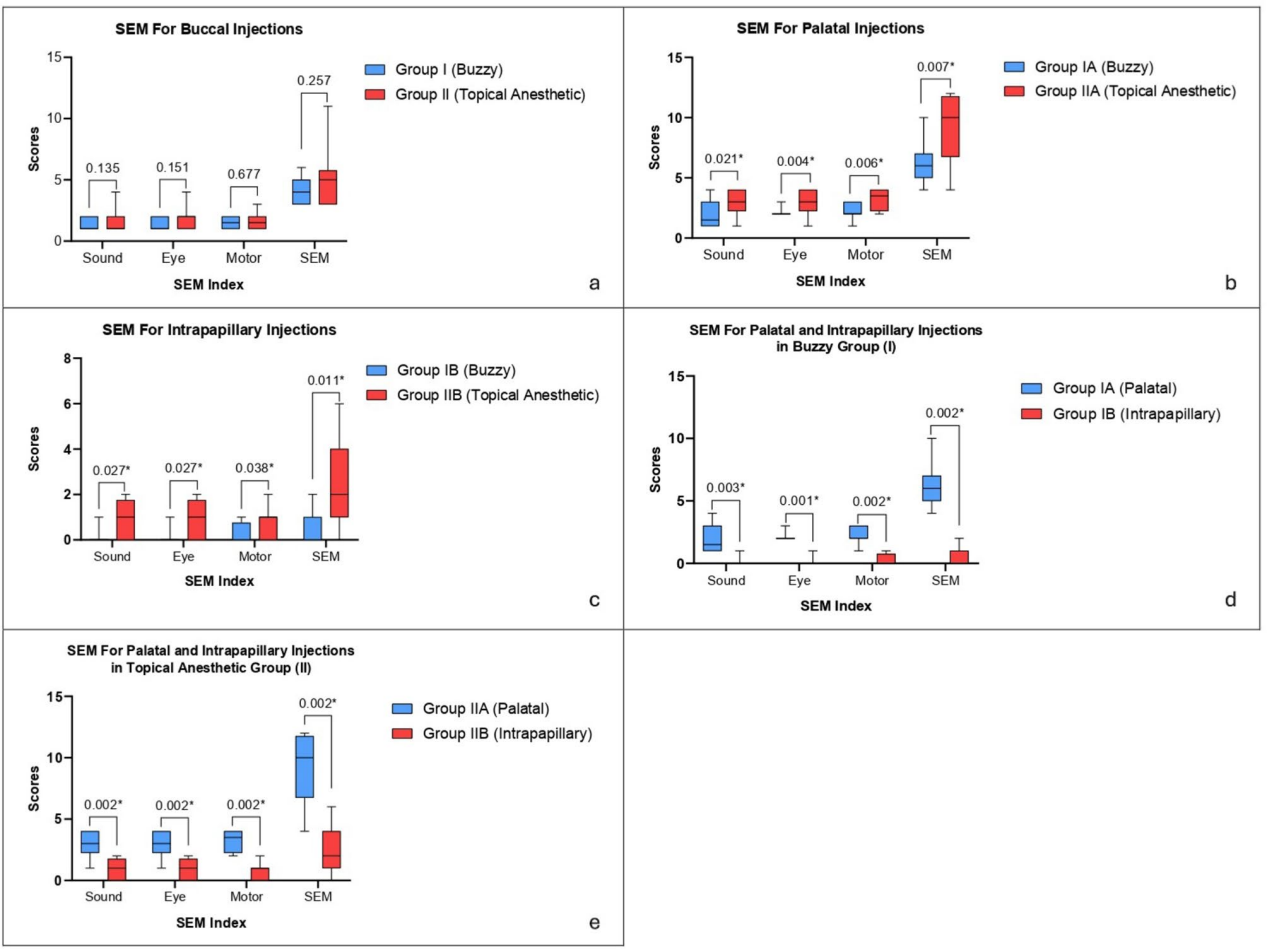
(**a**) Comparison of SEM scores between the two study groups during buccal injections (**b**) Comparison of SEM scores between the two study groups for palatal injections (**c**) Comparison of SEM scores between the two study groups for intrapapillary injections (**d**)Comparison of SEM scores between palatal and intrapapillary injections among patients received Buzzy. (**e**) Comparison of SEM scores between palatal and intrapapillary injections among patients received topical anesthetic gel

Regarding buccal infiltration injection, the comparison between test group I and control group II was evaluated using SEM (Fig. 1a) (Additional File 6) and VAS (Table 1). SEM showed no statistical significance in each parameter as well as the *total* score of the SEM scale, where p (*sound*) = 0.135, p (*eye*) = 0.151, p (*motor*) = 0.677, and p (*total* SEM) = 0.257. VAS reported no statistically significant scores in perceived pain in group I compared to group II where *p* = 0.106.

Regarding palatal infiltration injection, the comparison between the test group IA and the control group IIA was recorded using SEM Figure (1b) (Additional File 7) and VAS Table (1). SEM presented statistically significant lower pain scores regarding *sound*, *eyes*, *motor*, and *total* SEM evaluation in patients who applied Buzzy (group IA) in comparison with topical anesthetic gel (group IIA) during palatal injection (*p* = 0.021, *p* = 0.004, *p* = 0.006, *p* = 0.007 respectively).VAS showed that there was a statistically significant lower pain score in group IA compared to group IIA for palatal injections where *p* = 0.032.

Regarding intrapapillary infiltration injection, the comparison between the test group (IB) and the control group (IIB) was assessed using SEM Figure (1c) (Additional File 8) and VAS Table (1). SEM revealed statistically significant lower pain scores regarding *sound*, *eyes*,* motor*, and *total* SEM evaluation in patients who were given intrapapillary injection after applying Buzzy (IB) in comparison with topical anesthetic gel at the mucobuccal fold (IIB) (*p* = 0.027, *p* = 0.027, *p* = 0.038, *p* = 0.011 respectively). VAS showed statistically significant lower pain score in group IB compared to group IIB for intrapapillary injections where *p* = 0.016.

The comparison between the palatal (group IA) and the intrapapillary infiltration injections (group IB) in the test group was recorded using SEM (Fig. 1d) (Additional File 9) and VAS (Table 1). SEM showed statistically significant higher pain scores in IA regarding *sound*,* eyes*, *motor*,* and total* SEM evaluation (*p* = 0.003, *p* = 0.001, *p* = 0.002, *p* = 0.002 respectively). VAS presented statistically significant higher pain score in group IA compared to IB for palatal injections after the use of Buzzy where *p* = 0.009.

The comparison between palatal (group IIA) and intrapapillary infiltration injections (group IIB) in the control group was tested using SEM (Fig. 1e) (Additional File 10) and VAS (Table 1). SEM compared the pain perception results after applying topical anesthetic gel in the mucobuccal fold and on the hard palate in group IIA, and in the mucobuccal fold in group IIB. Statistically significant higher pain scores in IIA are presented regarding *sound*, *eyes*, *motor*, and *total* SEM evaluation where *p* = 0.002, *p* = 0.002, *p* = 0.002, *p* = 0.002 respectively. Regarding VAS, statistically significant higher pain scores are viewed in group IIA where *p* = 0.009.

All patients did not show any post-operative adverse effects after the dental procedures either after applying Buzzy or topical analgesic gel. None of the patients complained of lip biting or soft tissue injury after the visit.

**Table 1:** Comparison of visual analogue score (VAS) between the two study groups for different types of injections

Groups		Buccal injection	Palatal Injection (Subgroup A)	Intrapapillary Injection (Subgroup B)	W test# (*p* value)
**Buzzy Group (I)**	Mean ± SD	1.17 ± 1.24	3.50 ± 2.28	1.08 ± 0.99	2.615 (0.009*)
	Median	1.00	2.50	1.50	
	95% CI	0.0, 2.0	1.0, 6.0	0.0, 2.0	
	Min - Max	0.00–4.00	1.00–6.00	0.00–2.00	
	Mean Rank	21.42	9.46	9.38	
**Topical Anesthetic Group (II)**	Mean ± SD	2.08 ± 2.15	6.25 ± 2.96	2.92 ± 2.11	2.604 (0.009*)
	Median	2.00	6.00	2.50	
	95% CI	1.0, 2.0	4.0, 10.0	1.0, 5.0	
	Min - Max	0.00–10.00	1.00–10.00	0.00–6.00	
	Mean Rank	27.58	15.54	15.63	
**U test** (***p*** **value)**		1.615 (0.106)	2.147 (0.032*)	2.401 (0.016*)	

*Statistically significant difference at *p* value ≤ 0.05, #Wilcoxon Sign Rank test comparing palatal and intrapapillary injections within Buzzy Group (I) and Topical Anesthetic Group (II)

Figure 1 was designed by GraphPad Software (GraphPad Prism version 10.0.0 for Windows, Boston, Massachusetts USA).

Regarding the injection duration between the study groups, there was no statistically significant difference between group I and II during buccal, palatal and intrapapillary infiltration injection. (*p* = 0.947, *p* = 0.796, *p* = 0.971, respectively).

The comparison between the injection duration of the subgroups (palatal and intrapapillary infiltration) showed statistically significant lower scores in the palatal group in the test (*p* = 0.005) as well as the control group (*p* = 0.005).

## Discussion

Dental fear and anxiety negatively influence oral healthcare of children. Therefore, different methods that prevent patients from avoiding dental treatment and managing pain are important to every pediatric dentist. This study was conducted to evaluate the effectiveness of a vibratory unit with ice wings “Buzzy^®^” in pain reduction before administering buccal maxillary infiltration LA in comparison to the traditional topical anesthetic gel in children. Following buccal infiltration anesthesia, evaluation of the effectiveness of intrapapillary anesthesia in pain perception compared to the conventional palatal injection was also measured.

The results obtained partly rejected the null hypothesis. Pain perception during buccal infiltration anesthesia was comparable regarding Buzzy and conventional topical analgesic gel. On the other hand, Buzzy^®^ significantly decreased self-reported as well as observer- reported pain perception compared to conventional topical anesthetic gel prior to palatal and intrapapillary LA injection used for extraction in the maxillary arch.

Children 6 to 8 years old were included in this study since it has been proposed that this is the age at which signs of cognitive growth begin to develop [28]. The minimum age range was selected according to a study by Berghmans et al. [29] where they concluded that due to anxiety, children under five years old obtained higher scores on the VAS than those over six years and older. The maximum age range was chosen to avoid the normal primary molar shedding time which starts at 9 years, where extraction is not needed anymore [30]. 

Parallel design was adopted in this clinical trial to overcome the negative effect of LA injection and extraction on child’s behavior. Only extractions were performed to fulfill the study criteria in which pain during intrapapillary and palatal infiltration anesthesia are measured. Moreover, dental extractions added the benefit of finding other ways to reduce painful palatal LA.

Benaim et al. [31] stated that using self-report as the only indicator of pain severity in children who are in distress may confine scores in behavioral scales due to the minimal correlation between the results of the behavioral measures and the self-report. Hence, both objective (SEM) and subjective (VAS) pain scale were used in this study. SEM scale was selected to measure pain objectively since it has been shown to be accurate in measuring pain in children in numerous previous research [16, 32]. In this study, pain was assessed subjectively using a modified VAS that incorporated images of faces. The three pain intensity categories—mild, moderate, and severe—are the ones most frequently applied for pain assessment subjectively by the patient. Unlike what this classification would imply, pain does not appear to jump in discrete steps. Visual analogue scale was thus chosen to convey the concept of an underlying continuum of pain [33]. A good association between the Wong-Baker scale and this modified version of the VAS in children was established in a systematic review [34]. Nevertheless, studies have indicated that the Wong-Baker scale might exaggerate pain because fearful kids who aren’t in pain might not choose a happy face [34]. 

Pain perception during LA administration is influenced by four factors: the anesthetic’s pharmacologic characteristics, the tools utilized, the environmental circumstances, and the injection technique. Optimizing all four variables reduces patient suffering [35]. This study emphasizes on the equipment used to alleviate injection pain. The insertion of the needle during administration of LA, causes large diameter, myelinated A-delta fibers to create intense, pricking first pain. The added pressure from the fluid during the act of injection activates unmyelinated C-fibers by tissue distention, creating dull, diffuse second pain [35]. 

The proposed mechanism of action of Buzzy– as cold vibratory stimulant- unites the benefits of less painful injection through cold analgesia, tactile stimulation, and distraction without the use of medications or medical waste. The brain is only capable of identifying one stimulus at a time, whether painful or tactile. The initial stimulus that reaches the nervous system is vibration. It reduces pain, thus supporting the “Gate Control” theory of Melzack and Wall [5]. This is due to the activation of the large diameter nerve fibers responsible for touch and vibration stimuli. On one hand, the device’s vibration component stimulates the rapid, non-noxious motion-sensitizing A-beta fibers, which ultimately blocks the afferent pain-receptive A-delta fibers. On the other hand, the cold component activates the C fibers that also inhibits the A-delta pain signal when administered before the pain stimulus [36]. The device was found to be applicable for dental use in all children.

A study by Köse et al. [37]reported that the cartoon-assisted endoscopic preparation package was successful in lowering the fear and anxiety that children experience during the procedure. Hence, it is believed that the material’s colorful and animated design and bee-like shape will help children accept it in the clinical setting. Children who undergo several dental visits will anticipate the prick pain after the conventional topical gel. Altering the material to be used before LA by using a device like Buzzy, may prevent the anticipation on the long run.

According to Kohli et al. [38], a topical benzocaine-based gel with a concentration of up to 20% is poorly absorbed into the circulatory system, is used only in small doses, and has a low potential for overdosing, unlike lidocaine based topical anesthetics.

4% articaine with a 1:100,000 epinephrine was the LA solution of choice in this study as it exhibits remarkable chemical and pharmacological characteristics that increase its lipid solubility and diffusion rate through tissues. It has been reported to achieve 1.5 higher level of anesthetic potency and 0.6 lower systemic toxicity than lidocaine in all clinical situations [39]. Furthermore, articaine is metabolized in the blood serum as well as the liver. Kurtzman [40]stated that articaine may be a good local anesthetic choice for extraction in infected areas.

The handbook by Soxman and Malamed [41]recommended to start any treatment to a patient with no previous dental experience with a maxillary buccal anesthesia as it is not relatively painful compared to other techniques and is thus a less traumatic first experience for the child. A comparison between Buzzy and topical gel revealed a lower median SEM score (SEM = 4) in the Buzzy group compared to the topical gel group (SEM = 5) which indicates that participants with Buzzy experienced less pain, suggesting a more favorable pain response. Comparison of VAS scores revealed that both groups experienced mild pain, where Buzzy and topical gel groups reported a median VAS score of 1 and 2, respectively. However, both scales showed insignificant differences, denoting that both techniques were effective in reducing needle prick pain. The study results come in agreement with that of AlHareky et al. [16] where the objective assessment showed same pain perception by children during maxillary infiltration anesthesia when applying vibratory external cold stimuli or topical gel. The difference in results regarding the subjective pain scores can be explained based on age range that was broader than our study. Quick-acting, non-invasive topical skin refrigerants provide a short-term local anesthetic alternative to topical gel [35]. 

When comparing Buzzy group to topical gel group in palatal infiltration anesthesia, the findings showed that Buzzy (median SEM = 6) reported less pain than topical gel (median SEM = 10), supporting the idea that the intervention with Buzzy was more effective in reducing pain perception. Similarly, VAS results indicated that the Buzzy group experienced mild pain (median VAS = 2.5), while topical gel reported a moderate level of pain (median VAS = 6). Comparably, statistically significant objective and subjective results favoring Buzzy in comparison to topical gel have been recorded.

Distraction has been successfully implemented to sidetrack kids and minimize the discomfort associated with venipuncture and intramuscular injections [18]. In the present study, not only the vibratory and cold stimuli, but also the bee shaped cartooned device may have had a positive impact on distracting children. Moreover, preference of Buzzy over topical anesthesia in palatal injection may be explained by the unpleasant taste and smell of the gel [8]. 

In a study of maxillary extractions [42], little difference in the effectiveness of buccal infiltration administered alone, or those administered in combination with palatal injections was reported. The cancellous nature of the palatal bone, together with the ten minutes waiting period allowed to achieve anesthesia, may have abolished pain perception on the palatal tissues [43]. This may indicate that buccal anesthesia spreads to the palate, starting an anesthetic action on the palatal tissues even prior to palatal injection. In this context, a study by Houzain et al. [44]showed superior results of buccal infiltration LA administered alone over buccal and palatal injections in maxillary primary molar extraction. Head anatomy may explain positive results in decreasing pain in palatal infiltration after using Buzzy. The maxillary nerve passes through the foramen rotundum into the pterygopalatine fossa where it connects with the pterygopalatine ganglion. The ganglion carries both sensory fibers from the maxillary nerve and other parasympathetic fibers. One of the branches of the ganglion is the greater palatine nerve that transmits sensory fibers from the maxillary nerve and supplies the palate. The greater and lesser palatine nerves originate from the inferior surface of pterygopalatine ganglion and run through the palatine canal [45]. On one hand, the pterygopalatine fossa lies deep to the infratemporal fossa and posterior to the maxilla on both sides of the skull, also between the pterygoid process and the maxillary tuberosity, near the orbital apex [46]. On the other hand, the zygomatic bones protrude laterally and create the cheek’s prominence, a part of the lateral wall, the orbit floor, and some parts of the temporal fossa and infratemporal fossa [47]. Given the proximity of both fossa and the zygomatic bone, Buzzy vibrations on the zygoma for 2 min may reach the greater palatine nerve.

The comparison between Buzzy and topical gel during intrapapillary infiltration revealed a noticeable difference in pain perception, where Buzzy (median SEM = 0) reported no pain, while topical gel (SEM = 2) mild pain. This indicates that intrapapillary injections resulted in a lower pain perception with Buzzy compared to the topical gel. Comparison of VAS scores revealed that both groups experienced mild pain, where Buzzy and topical gel groups reported a median VAS score of 1.5 and 2.5, respectively. Nevertheless, significant objective and subjective results favoring Buzzy in comparison to topical gel have been shown. The effect of Buzzy is expected to reach the whole buccal area, including the interdental papillae, whereas topical gel is confined to the buccal sulcus only.

The comparison between the palatal and intrapapillary injections using Buzzy showed that palatal infiltration group reported a higher pain perception (median SEM = 6) than intrapapillary (median SEM = 0). Participants in topical gel group, who received palatal injections reported greater pain (median SEM = 10) than intrapapillary injections (median SEM = 2). These findings suggest that palatal injections, regardless of the group, led to a higher pain perception compared to intrapapillary injections. With Buzzy, VAS showed that palatal and intrapapillary groups experienced mild pain, with median VAS scores of 1.5 and 2.5, respectively. This indicates a comparable level of pain perception across both types of injections in the Buzzy group. However, with topical gel palatal injection was associated with moderate pain (median VAS = 6), while the intrapapillary injection caused mild pain (median VAS = 2.5). Statistical significance was reported favoring intrapapillary LA showing less objective and subjective pain perception compared to palatal LA after using Buzzy as well as topical gel. Abundance of literature suggests equipment and methods for relieving palatal infiltration pain, but none have been without drawbacks [8]. The present study agrees with that of Sruthi et al. [8]where intrapapillary injection is equally effective as the conventional technique for palatal anesthesia for maxillary extractions, with statistically significant less pain experienced during the intrapapillary LA administration. Because of the close attachment of the mucoperiosteum with the bone, palatal infiltration is thought to be the most painful [48]. 

The injection duration of the buccal infiltration was the longest, followed by intrapapillary and the least being the palatal. The amount of local anaesthesia administered was associated with the duration. The more local anaesthesia applied, the longer it took. There was no difference in the duration between Buzzy and topical gel groups, as the duration is not related to the method of pre-anaesthesia.

## Limitations

The cold stimulus was a cause of discomfort for some patients. Nonetheless, palatal infiltration anaesthesia caused more pain when introducing local anaesthesia which might overcome the disadvantage of using Buzzy.

## Conclusions

1. Buzzy appears to be a simple, practical, and non-invasive way for administering topical dental anesthesia for children.
2. External cold and vibratory stimulants can decrease the pain experienced during maxillary buccal infiltration injection.
3. Although palatal infiltration recorded lower pain perception with Buzzy, intrapapillary injections were more effective in reducing pain perception.

## Clinical implications

This study has the potential to enhance the understanding of effective pain management techniques, ultimately improving the pediatric dental experience and long-term dental outcomes for children. It may also contribute to evidence-based recommendations for pediatric dental professionals, helping to refine standard practices for local anesthesia in children.

## Electronic supplementary material

Below is the link to the electronic supplementary material.

Supplementary Material 1

Supplementary Material 2

## Data Availability

The datasets used and analysed during the current study are available from the corresponding author on reasonable request.

## Abbreviations

SEM: Sound eye motor
LA: Local anesthesia
VAS: Visual analog scale
