# Supplementary material for: Effectiveness of cold vibratory stimuli on pain perception governing infiltration anesthesia in the maxillary arch in children: a randomized controlled clinical trial
Source: BMC Oral Health. 2025 Jun 3;25:900. doi: 10.1186/s12903-025-06170-4 (PMC12135220; doi:10.1186/s12903-025-06170-4)
Supplement: Supplementary file 1 — Supplementary Material 1 [file 12903_2025_6170_MOESM1_ESM.docx]

Additional File 1

**ASA physical status classification**

| **ASA grade** |
| --- |
| I Normal healthy patient |
| II Patient with mild systemic disease |
| III Patient with severe systemic disease |
| IV Patient with severe systemic disease that is constant threat to life |
| V Moribund patient who is not expected to survive without the operation |
| VI Declared brain-dead patient whose organs are being removed for donor purposes |

Additional File 2

**Frankl behavior rating scale**

| **Rating** | **Behavior** | **Mild** **discomfort** |
| --- | --- | --- |
| **1** | Definitely  Negative | Refusing to play game, crying forcefully or fearfully, or any other overt evidence of extreme negativism |
| **2** | Negative | Reluctance to playing, uncooperative behaviour, and some evidence of negative attitude that is not pronounced |
| **3** | Positive | Acceptance of playing, willingness to comply with the dentist, cooperative behaviour |
| **4** | Definitely  Positive | Good rapport with the dentist, interested in the environment, laughing, and enjoying the situation |
|  |  |  |


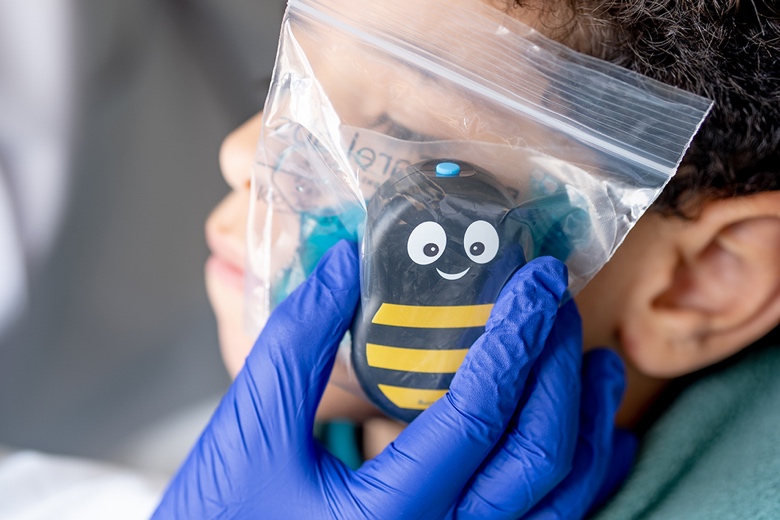


Additional File 3: Buzzy placed against the zygomatic arch and switched on.

Additional File 4

**SEM scale for the pain assessment from children’s behavior.**

|  | **Comfort or Pain Level** | | | |
| --- | --- | --- | --- | --- |
| **Observation** | **1. Comfort** | **2. Mild Discomfort** | **3. Moderately Painful** | **4. Painful** |
| Sounds | No sounds indicating pain | Nonspecific sounds; possible pain indication | Specific verbal complaints, e.g., "OW", raises voice | Verbal complaint indicates intense pain, e.g., screams, sobbing |
| Eyes | No eye signs of discomfort | Eyes wide, show of concern, no tears | Watery eyes, eyes flinching | Crying, tears running down face |
| Motor | Hands relaxed; no apparent body tenseness | Hands show some distress or tension; grasps chair due to discomfort, muscular tension | Random movement of arms or body without aggressive intention of physical contact, grimace, twitch | Movement of hands to make aggressive physical contact,e.g., punching, pulling head away |

Additional File 5

**Visual Analogue Scale**


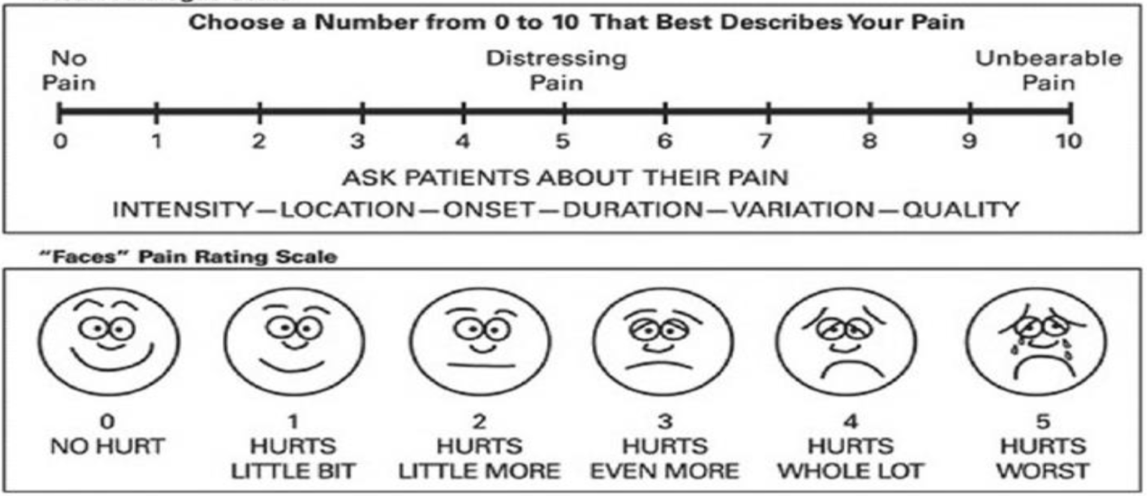


Additional File 6

**Comparison of SEM scores between the two study groups during buccal injections**

|  | | Group I  (n=24) | Group II  (n=24) | U Test  (*p* value) |
| --- | --- | --- | --- | --- |
| Sound | Median | 1.0 | 1.0 | 1.495  (0.135) |
|  | 95% CI | 1.0, 2.0 | 1.0, 2.0 |  |
|  | Min - Max | 1.0 – 2.0 | 1.0 – 4.0 |  |
|  | Mean Rank | 21.92 | 27.08 |  |
| Eye | Median | 1.0 | 2.0 | 1.436  (0.151) |
|  | 95% CI | 1.0, 2,0 | 1.0, 2.0 |  |
|  | Min - Max | 1.0 – 2.0 | 1.0 – 4.0 |  |
|  | Mean Rank | 21.94 | 27.06 |  |
| Motor | Median | 1.50 | 1.50 | 0.417  (0.677) |
|  | 95% CI | 1.0, 2.0 | 1.0, 2.0 |  |
|  | Min - Max | 1.0 – 2.0 | 1.0 – 3.0 |  |
|  | Mean Rank | 23.75 | 25.25 |  |
| SEM | Median | 4.0 | 5.0 | 1.133  (0.257) |
|  | 95% CI | 3.0, 5.0 | 3.0, 5.0 |  |
|  | Min – Max | 3.0 – 6.0 | 3.0 – 11.0 |  |
|  | Mean Rank | 22.29 | 26.71 |  |

95% CI: Confidence Interval, U test: Mann Whitney U test

**Figure 1**: (a) Comparison of SEM scores between the two study groups during buccal injections.

Additional File 7

**Comparison of SEM scores between the two study groups for palatal injections**

|  | | Group IA  (n=12) | Group IIA  (n=12) | U Test  (*p* value) |
| --- | --- | --- | --- | --- |
| Sound | Median | 1.50 | 3.0 | 2.310  (0.021*) |
|  | 95% CI | 1.0, 3.0 | 2.0, 4.0 |  |
|  | Min - Max | 1.0 – 4.0 | 1.0 – 4.0 |  |
|  | Mean Rank | 9.29 | 15.71 |  |
| Eye | Median | 2.0 | 3.0 | 2.862  (0.004*) |
|  | 95% CI | 2.0, 2.0 | 2.0, 4.0 |  |
|  | Min – Max | 2.0 – 3.0 | 1.0 – 4.0 |  |
|  | Meana Rank | 8.75 | 16.25 |  |
| Motor | Median | 2.0 | 3.50 | 2.727  (0.006*) |
|  | 95% CI | 2.0, 3.0 | 2.0, 4.0 |  |
|  | Min – Max | 1.0 – 3.0 | 2.0 – 4.0 |  |
|  | Mean Rank | 8.75 | 16.25 |  |
| SEM | Median | 6.0 | 10.0 | 2.710  (0.007*) |
|  | 95% CI | 5.0, 7.0 | 6.0, 12.0 |  |
|  | Min - Max | 4.0 – 10.0 | 4.0 – 12.0 |  |
|  | Mean Rank | 8.63 | 16.38 |  |

*Statistically significant difference at *p* value≤0.05, U test: Mann Whitney U test

**Figure 1**: (b) Comparison of SEM scores between the two study groups for palatal injections.

Additional File 8

**Comparison of SEM scores between the two study groups for intrapapillary injections**

|  | | Group IB  (n=12) | Group IIB  (n=12) | U Test  (*p* value) |
| --- | --- | --- | --- | --- |
| Sound | Median | 0.0 | 1.0 | 2.215  (0.027*) |
|  | 95% CI | 0.0, 0.0 | 0.0, 2.0 |  |
|  | Min - Max | 0.0 – 1.0 | 0.0 – 2.0 |  |
|  | Mean Rank | 9.75 | 15.25 |  |
| Eye | Median | 0.0 | 1.0 | 2.215  (0.027*) |
|  | 95% CI | 0.0, 0.0 | 0.0, 2.0 |  |
|  | Min – Max | 0.0 – 1.0 | 0.0 – 2.0 |  |
|  | Mean Rank | 9.75 | 15.25 |  |
| Motor | Median | 0.0 | 1.0 | 2.073  (0.038*) |
|  | 95% CI | 0.0, 1.0 | 0.0, 1.0 |  |
|  | Min – Max | 0.0 – 1.0 | 0.0 – 2.0 |  |
|  | Mean Rank | 9.88 | 15.13 |  |
| SEM | Median | 0.0 | 2.0 | 2.547  (0.011*) |
|  | 95% CI | 0.0, 1.0 | 1.0, 4.0 |  |
|  | Min – Max | 0.0 – 2.0 | 0.0 – 6.0 |  |
|  | Mean Ranak | 8.96 | 16.04 |  |

*Statistically significant difference at *p* value≤0.05, U test: Mann Whitney U test

**Figure 1**: (c) Comparison of SEM scores between the two study groups for intrapapillary injections.

Additional File 9

**Comparison of SEM scores between palatal and intrapapillary injections among patients received Buzzy**

|  | | Group IA  (n=12) | Group IB  (n=12) | W test  (*p* value) |
| --- | --- | --- | --- | --- |
| Sound | Median | 1.50 | 0.0 | 2.992  (0.003*) |
|  | 95% CI | 1.0, 3.0 | 0.0, 0.0 |  |
|  | Min - Max | 1.0 – 4.0 | 0.0 – 1.0 |  |
|  | Mean Rank | 9.29 | 9.75 |  |
| Eye | Median | 2.0 | 0.0 | 3.213  (0.001*) |
|  | 95% CI | 2.0, 2.0 | 0.0, 0.0 |  |
|  | Min – Max | 2.0 – 3.0 | 0.0 – 1.0 |  |
|  | Mean Rank | 8.75 | 9.75 |  |
| Motor | Median | 2.0 | 0.0 | 3.134  (0.002*) |
|  | 95% CI | 2.0, 3.0 | 0.0, 1.0 |  |
|  | Min – Max | 1.0 – 3.0 | 0.0 – 1.0 |  |
|  | Mean Rank | 8.75 | 9.88 |  |
| SEM | Median | 6.0 | 0.0 | 3.083  (0.002*) |
|  | 95% CI | 5.0, 7.0 | 0.0, 1.0 |  |
|  | Min – Max | 4.0 – 10.0 | 0.0 – 2.0 |  |
|  | Mean Rank | 8.63 | 8.96 |  |

*Statistically significant difference at *p* value≤0.05, W test: Wilcoxson Sign Rank test

**Figure 1**: (d) Comparison of SEM scores between palatal and intrapapillary injections among patients received Buzzy.

Additional File 10

**Comparison of SEM scores between palatal and intrapapillary injections among patients received topical anesthetic gel**

|  | | Group IIA  (n=12) | Group IIB  (n=12) | W test  (*p* value) |
| --- | --- | --- | --- | --- |
| Sound | Median | 3.0 | 1.0 | 3.108  (0.002*) |
|  | 95% CI | 2.0, 4.0 | 0.0, 2.0 |  |
|  | Min - Max | 1.0 – 4.0 | 0.0 – 2.0 |  |
|  | Mean Rank | 15.71 | 15.25 |  |
| Eye | Median | 3.0 | 1.0 | 3.082  (0.002*) |
|  | 95% CI | 2.0, 4.0 | 0.0, 2.0 |  |
|  | Min - Max | 1.0 – 4.0 | 0.0 – 2.0 |  |
|  | Mean Rank | 16.25 | 15.25 |  |
| Motor | Median | 3.50 | 1.0 | 3.086  (0.002*) |
|  | 95% CI | 2.0, 4.0 | 0.0, 1.0 |  |
|  | Min - Max | 2.0 – 4.0 | 0.0 – 2.0 |  |
|  | Mean Rank | 16.25 | 15.13 |  |
| SEM | Median | 10.0 | 2.0 | 3.064  (0.002*) |
|  | 95% CI | 6.0, 12.0 | 1.0, 4.0 |  |
|  | Min - Max | 4.0 – 12.0 | 0.0 – 6.0 |  |
|  | Mean Rank | 16.38 | 16.04 |  |

*Statistically significant difference at *p* value≤0.05, W test: Wilcoxson Sign Rank test

**Figure 1**: (e) Comparison of SEM scores between palatal and intrapapillary injections among patients received topical anesthetic gel.
